# The Surgical Complications and Sequels of Typhoid Fever

**Published:** 1898-12

**Authors:** 


### Books and Magazines.

---

THE SURGICAL COMPLICATIONS AND SEQUELS OF TYPHOID FEVER. By William W. Keen, M. D., LL. D., Professor of Surgery and Clinical Surgery, Jefferson Medical College, etc., etc. Based upon Tables of 1700 Cases, Compiled by the Author and by Thomas S. Wescott, M. D., Instructor in Diseases of Children, University of Pennsylvania, etc., with a Chapter on the Ocular Complications of Typhoid Fever, by George E. De Schweinitz, A. M., M. D., Professor of Ophthalmology, Jefferson Medical College, etc., and as an Appendix, The Toner Lecture No. V. W. B. Saunders, Philadelphia. 1898. Pages, 385. Price, cloth, $3, net.

This monograph does credit to its distinguished author, now perhaps the most eminent American surgeon. It had its origin in two notable lectures. On February 17th, 1876, the author delivered the Fifth Toner Lecture, "On the Surgical Complications and Sequels of Typhoid Fever," and fully brought the subject down to that date, and twenty years later being invited to deliver the Shattuck lecture before the Massachusetts Medical Society, he chose the same subject, and collected the literature, bringing the subject completely up to date. One must read the work to fully appreciate its value, as but few have an idea of the extent and importance of the subject. The work is absolutely complete and leaves nothing further to be said upon the subject at this time. Dr. Wescott has collected and compiled the literature, and Dr. Schweinitz written the chapters upon the ocular complications. The work is highly recommended.

